# Nebulised mesenchymal stem cell derived extracellular vesicles ameliorate *E. coli* induced pneumonia in a rodent model

**DOI:** 10.1186/s13287-023-03385-6

**Published:** 2023-06-06

**Authors:** Hector Gonzalez, Sean McCarthy, Claire Masterson, Declan Byrnes, Ignacio Sallent, Emma Horan, Stephen J. Elliman, Gabriele Vella, Adriele Prina-Mello, Johnatas D. Silva, Anna D. Krasnodembskaya, Ronan MacLoughlin, John G. Laffey, Daniel O’Toole

**Affiliations:** 1 REMEDI at CÚRAM Centre for Medical Device Research, University of Galway, Galway, Ireland; 2 Orbsen Therapeutics, IDA Business Park, Dangan, Galway, Ireland; 3 Translational Medicine Institute, Trinity College Dublin, Dublin, Ireland; 4 Wellcome-Wolfson Institute for Experimental Medicine, School of Medicine Dentistry and Biomedical Sciences, Queen’s University Belfast, Belfast, UK; 5 Aerogen, IDA Business Park, Dangan, Galway, Ireland

**Keywords:** Pneumonia, Acute respiratory distress syndrome, Mesenchymal stem cells, Nebulisation, Extracellular vesicles

## Abstract

**Background:**

Mesenchymal stem cell (MSC) derived extracellular vesicles (EVs) have been proposed as an alternative to cell therapy, creating new possible delivery modalities such as nebulisation. We wished to investigate the therapeutic potential of directly nebulised MSC-EVs in the mitigation of *Escherichia* *coli*-induced pneumonia.

**Methods:**

EV size, surface markers and miRNA content were assessed pre- and post-nebulisation. BEAS2B and A459 lung cells were exposed to lipopolysaccharide (LPS) and treated with nebulised bone marrow (BM) or umbilical cord (UC) MSC-EVs. Viability assays (MTT) and inflammatory cytokine assays were performed. THP-1 monocytes were stimulated with LPS and nebulised BM- or UC-EVs and phagocytosis activity was measured. For in vivo experiments, mice received LPS intratracheally (IT) followed by BM- or UC-EVs intravenously (IV) and injury markers assessed at 24 h. Rats were instilled with *E. coli* bacteria IT and BM- or UC-EVs delivered IV or by direct nebulisation. At 48 h, lung damage was assessed by physiological parameters, histology and inflammatory marker presence.

**Results:**

MSC-EVs retained their immunomodulatory and wound healing capacity after nebulisation in vitro. EV integrity and content were also preserved. Therapy with IV or nebulised MSC-EVs reduced the severity of LPS-induced lung injury and *E. coli*-induced pneumonia by reducing bacterial load and oedema, increasing blood oxygenation and improving lung histological scores. MSC-EV treated animals also showed lower levels of inflammatory cytokines and inflammatory-related markers.

**Conclusions:**

MSC-EVs given IV attenuated LPS-induced lung injury, and nebulisation of MSC-EVs did not affect their capacity to attenuate lung injury caused by *E. coli* pneumonia, as evidenced by reduction in bacterial load and improved lung physiology.

**Supplementary Information:**

The online version contains supplementary material available at 10.1186/s13287-023-03385-6.

## Introduction

Acute respiratory distress syndrome (ARDS) remains a global concern with mortality rates of 30–45% and intensive care unit (ICU) prevalence of 10% [1]. Patients present with dyspnoea, chest pain and cyanosis, with bilateral pulmonary infiltration, oedema and hypoxemia [2]. In many ARDS patients, pneumonia is the underlying predisposing cause, with Gram-negative bacilli infections presenting the worst prognosis [3]. No specific treatment is available and current management remains supportive. Mesenchymal stem cells (MSCs) have antibacterial and immunomodulatory capacities and have emerged as a promising therapy in ARDS, where several pre-clinical studies have shown mitigation of inflammatory processes through modulation of immune cells [4–7], reduction of bacterial load and enhancement of tissue recovery [8, 9]. Despite promising preclinical results and demonstrated safety in humans, there remain challenges to be addressed for MSC therapy to become a reality in ARDS treatment. The need for large numbers of MSCs for human clinical applications is a logistical challenge, and cryopreservation methods are still inefficient [10]. Extracellular vesicles (EVs) derived from MSCs present an alternative to MSC administration with distinct biological and logistic advantages. In pre-clinical studies, EVs retained the capacity of MSCs themselves in mitigating inflammatory processes and reducing lung injury [5, 11], with apparent involvement of molecules such as short interfering RNA species miR-146 and mIR-181-5p. Large-scale manufacture of EVs can be conducted in bioreactors, reducing production costs by conserving time, reagents and materials. In addition, EVs can be stored without cryopreservants and remain stable for long periods without loss of therapeutic efficacy [12].

### Nebulisation: a new delivery route for MSCs products

Local administration has proved beneficial for the resolution of ARDS in preclinical studies [13–15]. However, direct administration of a fluid suspensions bolus can create physical damage to tissue already affected by infection [16].In addition, 70% of ARDS patients require mechanical ventilation [17], hindering local administration. While aerosols have been used in different lung pathologies for decades, newer technology vibrating mesh nebulisers have improved delivery and distribution throughout the airways [18]. In ARDS, vibrating mesh technology allows synchronising the delivery of the therapeutic to the ventilator pattern, maintenance of a closed circuit, no additional flow or pressures added to the circuit and enhances delivered dose while minimising waste of therapeutic. In addition, nebulisation of the product reduces the direct liquid damage caused to the alveolar parenchyma. Currently, pre-clinical ARDS studies are investigating the feasibility of nebulising different therapies such as corticoid [19], heparin [20], mucolytic agent [21] and surfactant [22] treatment with promising initial results.

We hypothesise that MSC-derived EVs retain therapeutic capacity after being nebulised into the lungs, offering a novel delivery route that can ameliorate lung injury arising from *Escherichia coli* (*E. coli*) pneumonia.

## Material and methods

### EV isolation and characterisation

#### EV isolation

BM-MSCs used in these experiments were isolated from healthy volunteers with informed consent under Galway University Hospital Clinical Research Ethics Committee approval 15/12. Bone marrow aspirate was diluted with media and plated as previously described [23]. Adherent cells were expanded until 80% confluent and further cultured to passage 3. At 70% confluence, cells were washed and cultured in serum-free medium for 24 h. Conditioned media (CM) was harvested and centrifuged at 4000× *g* for 20 min to discard cell debris and then at 100,000× *g* for 2 h and supernatant discarded. The pellet was washed with phosphate buffered saline (PBS) and centrifuged again at 100,000× *g* for 2 h. Supernatant was discarded, and pellets resuspended in 500µL of PBS and stored at − 80 °C until required.

UC-MSC EVs were provided by Orbsen Therapeutics Ltd. (Galway, Ireland) and were isolated from healthy volunteers with informed consent under Galway University Hospital Clinical Research Ethics Committee approval 59/12. CD362^+^ sorted UC-MSC CM expanded to P3 on the Quantum® Cell Expansion System (Terumo UK Ltd., Surrey, UK) [8]. CM underwent tangential flow filtration (TFF) via KrosFlo® KR2i (Repligen, Waltham, MA) using a 100 kDa filter. EVs were stored in PBS with PVP 5% and Trehalose 50 mM at − 80 °C.

### EV characterisation

#### Protein content

Protein concentrations of EV samples were measured using the Pierce bicinchoninic acid protein quantification (BCA) protein assay kit (Pierce, Thermo Fisher) as per manufacturer’s guidelines. A 12 point, twofold serial dilution of the BCA standard was performed with the highest point value at 2000 μg/mL. 25μL of pre and post nebulisation EV samples were added to a 96 well plate in triplicate and 150μL of the BCA assay solution was added to each well. The absorbance values were read in a VICTOR plate reader (Perkim Elmer, Whaltham, Massachusetts, USA) at a 550 nm wavelength and the protein concentrations of the samples was quantified against the standard curve.

#### miRNA content assessment

RNA was extracted using ‘miRNeasy’ Kit (Qiagen). Purity and concentration of the extraction were evaluated using a Nanodrop™ 2000 spectrometer (ThermoFisher Scientific Ltd). Reverse transcription and RT-PCR was performed using miRCURY LNATM miRNA PCR starter kit (Qiagen) following the manufacturer’s specifications. Non-nebulised and nebulised EV samples were screened for miRNA-146a-5p and miR-181-5p with the supplied primers using a StepOnePlus™ Real-Time PCR System (Applied Biosystems).

#### CFSE labelling of EV and transfer to lung cells

100 μL of PBS containing 4 × 10^8^ EV particles were incubated for 15 min at room temperature with 20μL of working concentration CFSE (CellTraceTM CFSE Cell Proliferation Kit; Invitrogen) prepared following manufacturer’s instructions. EVs were collected using an Exoeasy Maxi kit (Qusagen) and nebulised as previously described. Preparations were passed through EV isolation columns (Exoeasy Maxi kit, Quiagen) to collect supernatant and EVs separately. A549 lung cells were cultured with membrane marker PKH26 (Sigma) following the manufacturer’s protocol, cultured until 80% confluence was reached, and treated with either non-nebulised or nebulised CFSE labelled EVs or supernatant for 4 h. For microscopy, cells were washed with PBS, fixed using 4% PFA and nuclei were marked with DAPI. A total of 3 wells per treatment were imaged using the Olympus FluoView 1000 confocal laser scanning microscope at 60X magnification. Analysis was performed using imageJ (National Institute of Health, Bethesda, USA). For flow cytometry, cells were harvested, washed and resuspended in PBS. CFSE signal was measured using a BD Accuri C6 + flow cytometer (BD Biosciences). A minimum of 10,000 events was analysed and cells without staining served as negative controls and were used to discriminate between positive and negative populations using line gates for each marker. Data were analysed using FlowJo software (vX.07, FlowJo).

#### Size and surface markers

The presence, integrity and quantity of EVs were measured using nanotracking technology analysis (NTA) on a NanoSight NS500 system (Malvern Panalytical, Malvern, UK) in light scattering mode, following the EUNCL PCC-023 validation protocol and validated at EC level (www.euncl.eu) [24, 25]. Six separate 60 s videos were recorded for each sample and detection threshold was selected to ensure only distinct nano-objects were analysed. Surface markers were measured using a MACSPlex Exosome Kit (Miltenyi Biotec, Bergisch Gladbach, Germany) containing fluorescently labelled (FITC-PE) capture beads coupled to 37 exosomal surface epitopes and 2 isotope controls (in detail: CD3, CD4, CD19, CD8, HLA-DR, CD56, CD105, CD2, CD1c, CD25, CD49e, ROR1, CD209, CD9, SSEA-4, HLA-ABC, CD63, CD40, CD62P, CD11c, CD81, MCSP, CD146, CD41b, CD42a, CD24, CD86, CD44, CD326, CD133-1, CD29, CD69, CD142, CD45, CD31, REA control, CD20, CD14, mIgG1 control). Briefly, 15μL of beads were added to 120μL of buffer or sample, including a total of 10^9^ EVs, and the complex was then incubated on a rotor overnight at 4 °C. After the incubation and washing steps, a cocktail of APC fluorescent antibodies against tetraspanins (CD9, CD63 and CD81) was added allowing the detection of bead-bound EVs and set on the rotor for 1 h at room temperature. After washing, samples were detected using BD FACSCanto™ flow cytometer (BD Bioscience, Franklin Lakes, NJ, USA). Median background values of buffer control were subtracted, and samples were normalised to the median fluorescence intensity of tetraspanins.

### In vitro* EV therapeutic evaluation*

***EV nebulisation.*** Nebulisation was performed using an Aerogen Solo vibrating mesh nebuliser (Aerogen Ltd., Galway, Ireland). The nebuliser was placed over a sterile 50 mL collection tube in a tissue culture biosafety cabinet and secured with Parafilm. BM-MSC EVs and UC-MSC EVs were applied to the nebuliser reservoir and the aerosol allowed to condense in the collection tube.

#### Scratch wound assay

A549 lung epithelial cells were seeded to 24-well plates at 10^5^ cells/cm^2^ and 24 h later a single scratch wound introduced per well with a 1000 µL pipette tip. Wells were aspirated, rinsed with PBS and re-fed with 300 µL of MEM-α medium followed by vehicle (PBS), 2 × 10^9^/mL unprocessed BM-EVs or UC-EVs, or 2 × 10^9^/mL nebulised BM-EVs or UC-EVs. At pre-defined timepoints up to 24 h, scratch wounds were imaged by light microscopy and wound width assessed through measurement of pixel distance across the wound.

#### Cell viability assay

MTT reagent (5 mg/mL) (3-(4, 5-dimethylthiazol-2-yl)-2,5-diphenyltetrazolium bromide; Sigma-Aldrich Ltd., Arklow, Ireland) reconstituted in culture medium was used to evaluate cell viability and proliferation in BEAS2B cell monolayers randomised to receive *E. coli* lipopolysaccharide (LPS) (250 ng/mL) or vehicle (PBS) activation. Then they received 2 × 10^9^/mL of BM-EV or UC-EV preparations, nebulised or non-nebulised. After treatment, media was collected for cytokine content analysis, cells washed with PBS, and incubated with MTT reagent for 3 h at 37 °C in a humidified cell culture incubator. MTT reagent was replaced with dimethyl sulfoxide and absorbance readings measured using the Varioskan™ Flash microplate reader (Thermo Fisher Ltd.) at 595 nm wavelength. Cell viability was presented as a percentage relative to uninjured control.

#### Cytokine production

BEAS2B cell monolayers randomised to receive *E. coli* LPS (250 ng/mL) or vehicle were treated with vehicle or 2 × 10^9^/mL BM-EV or UC-EV preparations, nebulised or non-nebulised. After treatment, media were analysed for interleukin (IL)-8 and IL-1β cytokine concentration by enzyme linked immunosorbant assay (ELISA) as per manufacturer’s instructions (R&D Systems, Abingdon, UK).

#### Phagocytosis assay

THP-1 monocytes were seeded at 10^5^ cells/cm^2^ in 96-well plates and exposed to phorbol myristate acetate (PMA) (1 µg/mL) for 72 h to cause differentiation to macrophages. LPS (100 ng/mL) was added for a further 24 h. Macrophages were washed and treated with vehicle (PBS), 2 × 10^9^/mL unprocessed BM-EVs or UC-EVs, or 2 × 10^9^/mL nebulised BM-EVs or UC-EVs. After 2 h, fluorescently labelled zymosan particles (10 µg/mL) were added for 4 h and phagocytosis quantified through counting of engulfed particles inside macrophages under fluorescent microscopy. Two particles per cell was considered positive for phagocytosis.

### LPS-induced lung injury mouse model

We are adhering to the ARRIVE guidelines (http://www.nc3rs.org.uk/page.asp?id=1357) for the reporting of animal experiments. Work was approved by the Animal Welfare Ethical Review Body of Queen's University Belfast, in accordance with UK Animals (Scientific Procedures) Act 1986. Male C57BL/6 mice (8–12 weeks old; Envigo, Blackthorn, UK) were used. Mice were anaesthetised with xylazine (0.25 mg kg^−1^) and ketamine (0.025 mg kg^−1^) IP and 2 mg kg^−1^ *E. coli* LPS (O111:B4; Merck, Darmstadt, Germany) in 30 μL PBS instilled to the trachea under direct vision. 4 h later, 5 × 10^8^ BM-EV, 5 × 10^8^ UC-EV or 5 × 10^9^ UC-EV in 30μL PBS or vehicle were delivered via tail vein. Mice were allowed to recover and returned in their cages. 24 h later mice were sacrificed and broncheoalveolar lavage (BAL) performed for analysis as below.

### E. coli induced pneumonia rat model

Work was approved by the University of Galway institutional ethical body and conducted under license from the Health Products Regulatory Agency Ireland. Specific-pathogen-free adult male Sprague Dawley rats (weight 350–450 g) were used. A schematic of the nebulisation and ventilation set-up and the experimental protocol is depicted in Additional file 1: Fig. S1.

#### E. coli and EV administration

Animals were anaesthetised by inhalational isoflurane and following confirmation of anaesthesia depth, 1.5 × 10^9^ *E. coli* (serotype O9 K30 H10; Central Public Health Laboratory, London, UK) colony-forming units (CFU) in a 300μL PBS suspension was instilled into the trachea under direct vision. Rats remained under anaesthesia for 1 h and vehicle (PBS), 10^9^ BM-EVs or 10^9^ UC-EVs in 300μL PBS was administrated either IV or via an Aerogen Solo nebuliser on the FlexiVent small animal ventilator (Scireq, Montreal, Canada). Ventilation was a respiratory rate of 90 breaths/minute, FiO_2_ 0.21, tidal volume of 7 mL/kg, and positive end-expiratory pressure (PEEP) of 2 cm H_2_O. Afterwards, animals were allowed to recover and returned in their cages.

#### Assessment of lung injury

48 h after injury induction rats were anaesthetised using subcutaneous administration of 75 mg kg^−1^ ketamine (Chanelle, Loughrea, Ireland) and 0.5 mg kg^−1^ medetomidine (Vetoquinol, St.-Anne Lure, France). A tracheostomy was performed, and carotid artery access gained. Anaesthesia was maintained during the procedure through administrating Alfaxan IV (Jurox, Crawly, UK). Animals were mechanically ventilated and blood pressure and peak airway pressure were measured continuously. Static lung compliance and arterial blood gas analysis were measured after 20 min and repeated after 15 min of ventilation with 100% O_2_. Animals were then sacrificed by exsanguination under anaesthesia.

#### Ex vivo* analysis*

The heart–lung block was isolated and BAL was performed. The top left lobe was isolated for wet:dry analysis and right lobe was inflated with and fixed in 4% PFA for histological processing. Bacterial load was measured by serial dilution plating of BAL on Brilliance UTI agar (Fannin, Galway, Ireland) plates, incubated for 24 h at 37 °C, and counting of CFUs. Total BAL cells were counted using a haemocytometer and inflammatory cytokine levels were measured from a random selection of BAL samples using a 23-cytokines multiplex assay (Bio-Rad, Naas, Ireland) or standard ELISA (R&D systems).

### Statistical analysis

Data were analysed using GraphPad PRISM (GraphPad Software, San Diego, CA). Data distribution was tested for normality using Kolmogorov–Smirnov test and analysed by one-way ANOVA, with post hoc testing using Dunnett’s test with the vehicle group as the single comparison group or with Student–Newman–Keuls between group comparisons as appropriate. A two-tailed *p*-value of < 0.05 was considered significant.

## Results

### Intravenous delivery of EVs ameliorates LPS-induced lung injury

**Fig. 1:**
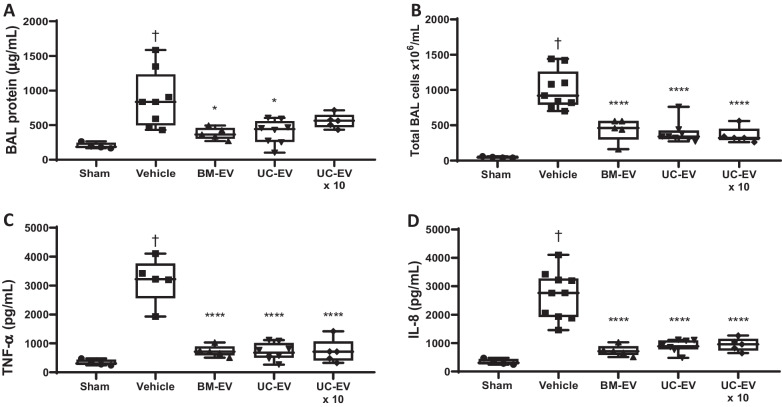
MSC-EVs delivered IV in a mouse LPS ARDS model. LPS (1 mg kg^−1^) was given to mice intratracheally followed by MSC-EVs IV. 24 h later BAL was performed and total protein measured by BCA assay **(A)**, total infiltrating cells were counted by staining and haemocytometry **(B)** and cytokine ELISAs were performed to quantify TNF-α **(C)** and IL-8 **(D)** concentrations in BAL. Sham N = 4, Vehicle N = 8, BM-EVs N = 5, UC-EVs N = 8, 10 × UC-EVs N = 5. † *p* < 0.05 wrt sham. *p < 0.05 wrt LPS with vehicle

In a mouse intratracheal model of LPS-induced lung injury, increased levels of total protein, indicative of lung permeability, were observed in the BAL at 24 h, and this was significantly ameliorated by IV administration of either BM-EV or UC-EV (Fig. 1A). Interestingly, the increased 5 × 10^9^ dose did not reach significance in this parameter. Total infiltrating immune cells in the BAL was significantly reduced by both types of EV therapy (Fig. 1B), as were BAL concentrations of the inflammatory cytokines TNF-α (Fig. 1C) and IL-8 (Fig. 1D).

### Effect of nebulisation on EV miRNA content and target cell fusion

**Fig. 2:**
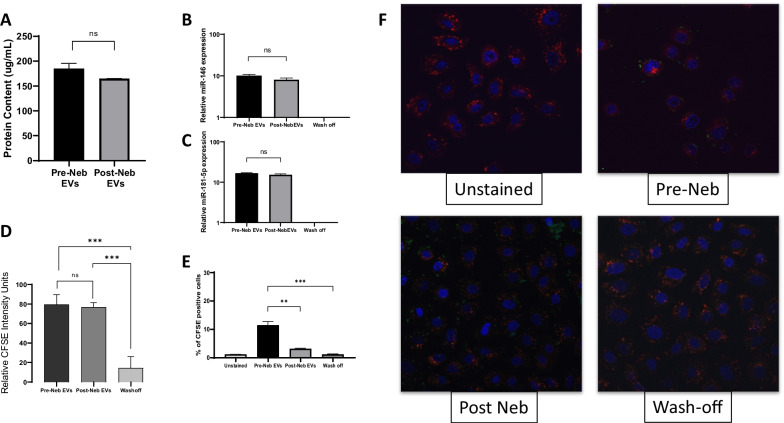
EV payload and integration with target cells pre- and post-nebulisation. UC-EV were passed through a nebuliser and total protein (**A**), mIR-146 **(B)** and miR-181-5p **(C)** quantified by QPCR. The mean intensity of a CSFE-label delivered by EVs to target lung epithelial cells was quantified by flow cytometry **(D)**. The percentage of cells in a lung epithelial monolayer that was positive for CFSE label was also assessed with EVs pre- and post-nebulisation **(E)** and representative images selected **(F)**. Pre Neb-EVs N = 3, Post Neb-EVs N = 3, Wash-off N = 3. **p < 0.01. ***p < 0.001. ns = no significant difference

UC-MSC EV preparation had similar total protein concentrations pre- and post-nebulisation (Fig. 2A). In analysis of interfering short miRNA which are known effectors of EV function, mIR-146 (Fig. 2B) and miR-181-5p (Fig. 2C) were shown to be equally abundant pre- and post-nebulisation, with the reverse washout of the nebuliser showing no signal. When CFSE labelled EVs were added to lung epithelial cells, flow cytometry indicated that nebulisation of the EVs did not significantly alter the mean CSFE fluorescence delivered to each cell (Fig. 2D). However, in image analysis of fixed monolayers, diminished CSFE positive cell numbers were observed in the lung epithelial monolayer (Fig. 2E, representative images Fig. 2F).

### Effect of nebulisation on EV size and surface marker

**Fig. 3:**
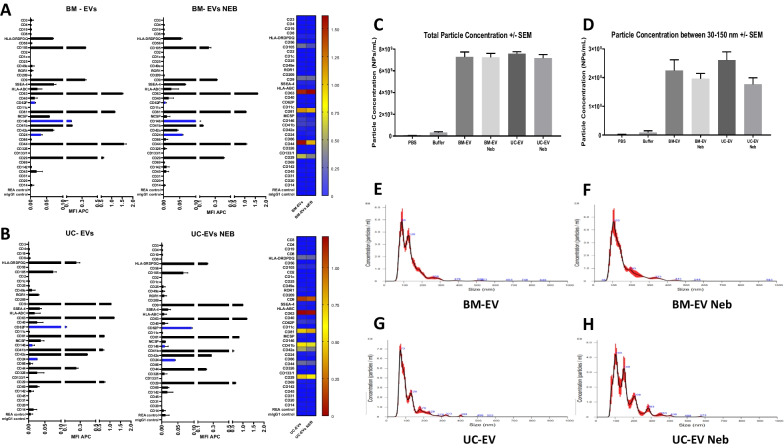
Nanotracker and surface marker analysis of EVs pre- and post-nebulisation. BM-EV (**A**) and UC-EV (**B**) samples were analysed by MACSPlex Exosome Kit surface marker antibody panel and flow cytometry, with pre- (left side) and post- (right side) nebulisation samples compared by heat map. In nanotracker analysis, total number of particles was established for each EV type pre- and post-nebulisation **(C)** and in the exosome size range **(D)**. Particle size distributions were also constructed for BM-EV pre- **(E)** and post- **(F)** nebulisation and for UC-EV pre- **(G)** and post- **(H)** nebulisation. N = 6

EVs derived from BM-MSCs and UC-MSCs were characterised following the ‘International Society for Extracellular Vesicles’ guidelines [26]. EVs from both cell sources presented the characteristic tetraspanins CD81, CD63 and CD9, among other markers for MSC-derived EVs such as CD44, CD29 and CD105 (Fig. 3A, B). Nebulisation resulted in no significant reduction of these markers on EV membranes (Fig. 3A, B). NTA analysis of BM-EVs showed a concentration of 7.28 × 10^9^ particles/mL on the whole measurable range (30-1000 nm) (Fig. 3C) and 2.2 × 10^8^ particles/mL when only small particles are considered in the analysis (30-150 nm) (Fig. 3D). UC-EVs showed a similar concentration on the whole measurable range of 7.57 × 10^8^ particles/mL (30-1000 nm) (Fig. 3C) and 2.2 × 10^8^ particles/mL when only small particles are considered in the analysis (30–150 nm) (Fig. 3D). Nebulisation of both EV preparations produced a non-significant reduction in the final concentration of EVs when the whole range (Fig. 3C) and small particles sizes (Fig. 3D) were analysed. BM-EVs had a mode distribution size of 94.53 nm with a clear higher concentration of particles around the 100 nm size (Fig. 3E) while UC-EVs had 71.85 nm mode size (Fig. 3F). In addition, nebulisation induced a change in the size distribution of the EV preparations, where nebulised BM-EVs increased their mode size distribution up to 106 nm (Fig. 3F) and UC-EVs to 118 nm (Fig. 3H).

### Effect of nebulisation on EV immunomodulatory activity

**Fig. 4:**
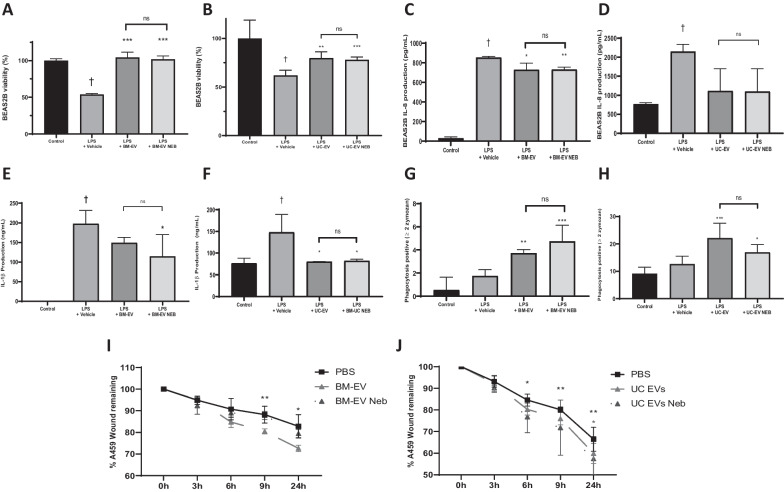
Pre- and post- nebulisation MSC-EV effects in in vitro lung cell injury models. BM- (**A**) and UC- (**B**) EVs were used directly or after nebulisation in a BEAS2B lung epithelial cell LPS injury model and viability assessed by MTT assay. IL-8 **(C + D)** and IL-1β **(E + F)** levels were quantified by ELISA in the same model with the same respective tissue sources of EVs. BM- **(G)** and UC- **(H)** EVs were applied to a zymosan-based macrophage phagocytosis assay and activity quantified by fluorescent microscopy. Finally, pre- and post-nebulisation BM- **(I)** and UC- **(J)** EVs were added to a lung epithelial cell scratch wound restitution model and remaining would size quantified intermittently by microscopy and image analysis.. Control N = 3–6, BM-EV N = 3–6, Neb BM-EVs N = 3–6, UC-EVs N = 3–6, Neb UC-EVs N = 3–6. † *p* < 0.05 wrt sham. *p < 0.05, **p0.01, ***p < 0.005, ****p < 0.001 wrt injury with vehicle. ns = no significant difference between IV and Neb delivery

BEAS2B cells had significantly reduced viability after exposure to LPS, and cells treated with BM- (Fig. 4A) or UC- (Fig. 4B) derived EVs significantly mitigated this injury compared with the vehicle group. Nebulisation of the BM-EVs (Fig. 4A) and UC-EVs (Fig. 4B) did not significantly modify the EV’s capacity to preserve cell viability after LPS exposure. BEAS2B exposed to LPS had significantly increased production of IL-8 and IL-1β pro-inflammatory cytokines in comparison with controls and BM-EVs or UC-EVs mitigated this increase of both IL-8 (Fig. 4C, E) and IL-1β (Fig. 4D, F) with no significant change in this effect observed after nebulisation of the EVs.

### Effect of nebulisation of EV on promotion of phagocytosis

Stimulation of macrophage-like THP-1 cells with LPS produced a non-significant increase in the phagocytic capacity of these cells. When cells were further treated with BM-EVs (Fig. 4G) and UC-EVs (Fig. 4H) there was an increased capacity to endocytose opsonised foreign particles. Nebulisation of the BM-EVs (Fig. 4G) and UC-EVs (Fig. 4H) did not diminish the capacity of EVs to promote the phagocytosis.

### Effect of nebulisation of EVs on wound healing capacity

Treatment of A459 monolayer scratch wounds with BM-EVs (Fig. 4I) or UC-EVs (Fig. 4J) accelerated the wound healing process when assessed at 9 h and 24 h after injury. Nebulisation of BM-EVs (Fig. 4I), but not UC-EVs (Fig. 4J), delayed the therapeutic effect of the EVs. As a multitude of MSC and EV derived factors can drive cell growth and proliferation, it is possible that BM and UC derived MSCs and EVs perform this function in slightly different ways, where one set of factors is more susceptible to degradation by the nebulisation process. This insight could be used to inform future clinical trials for specific pathologies such as oedema.

### Effect of EV therapy delivered by nebulisation on E. coli-induced pneumonia

**Fig. 5:**
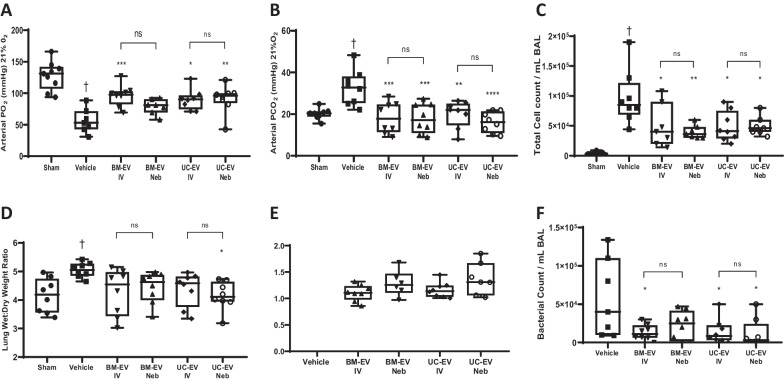
IV and nebulised EV effects on lung function in *E. coli* induced pneumonia. Rats received a bolus of *E. coli*, 1 h later were administered BM- or UC-EVs by IV or nebulisation route, and various physiological parameters assessed at 48 h. PO2 **(A)** and PCO2 **(B)** were assessed in arterial blood under 21% oxygen ventilation by blood-gas analyser. Total infiltrating leukocytes in the BAL were counted by differential staining and haemocytometer **(C)**, and the relative amount of fluid in the lung tissue was determined by weighing tissue sample when freshly harvested and dessicated **(D)**. Respiratory static compliance was assessed in lungs **(E)** and finally, BAL was plated to agar and bacterial load quantified through colony counting **(F)**. Control N = 4–9, Vehicle N = 7–8, BM-EV N = 6–8, Neb BM-EVs N = 6–8, UC-EVs N = 6–8, Neb UC-EVs N = 6–8. † *p* < 0.05 wrt sham. *p < 0.05, **p0.01, ***p < 0.005 wrt pneumonia with vehicle. ns = no significant difference between IV and Neb delivery

Rats treated with BM-EVs and UC-EVs presented higher arterial PO_2_ (Fig. 5A) and significantly lower PCO_2_ (Fig. 5B) than the vehicle group, with no significant difference between IV and nebuliser delivery. BAL isolated from those animals had a decrease in the total amount of cell infiltrate (Fig. 5C) in animals administered BM-EVs or UC-EVs with again no significant difference between IV or nebuliser delivery. In addition, EV groups had a reduction in the wet:dry ratio (Fig. 5D) with a corresponding increase in lung compliance in nebulised EVs (Fig. 5E), regardless of the cell source. BAL *E. coli* CFU was reduced after BM-EV or UC-EV therapy compared with the vehicle group, and delivery method did not modify this beneficial effect (Fig. 5F).

### Nebulised EV therapy mitigates lung structure damage in E. coli pneumonia

**Fig. 6:**
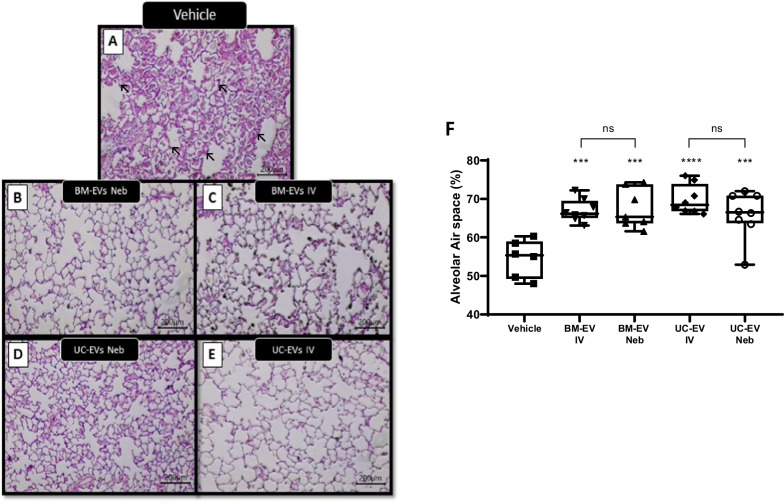
IV and nebulised EV effects on lung structure in *E. coli* induced pneumonia. Rats received a bolus of *E. coli*, 1 h later were administered BM- or UC-EVs by IV or nebulisation route, and at 48 h lungs were sectioned, stained and airspace calculated by stereology. Representative images of vehicle only **(A)**, BM-EV delivered by nebuliser **(B)**, BM-EV delivered IV **(C)**, UC-EV delivered by nebuliser **(D)**, and UC-EV delivered IV **(E)** are provided. Stereological analysis was used to graph average airspace in all images **(F)**. Arrows indicate alveolar thickening in vehicle animal. Vehicle N = 5, all other groups N = 8. **p0.01, ****p < 0.001 wrt pneumonia with vehicle. ns = no significant different between IV and Neb delivery

Intratracheal installation of *E. coli* produced alveolar wall thickening, infiltration, and atelectasis in animal lungs (Fig. 6A). Animals treated with nebulised BM-EVs (Fig. 6B), BM-EVs delivered IV (Fig. 6C), UC-EVs nebulised (Fig. 6D) or UC-EVs delivered IV (Fig. 6E) showed an increase in the alveolar air space compared with the non-treated animals without any significant difference between cell source or delivery modality (Fig. 6F).

### Nebulised EV therapy reduces inflammatory cytokines in E. coli pneumonia

**Fig. 7:**
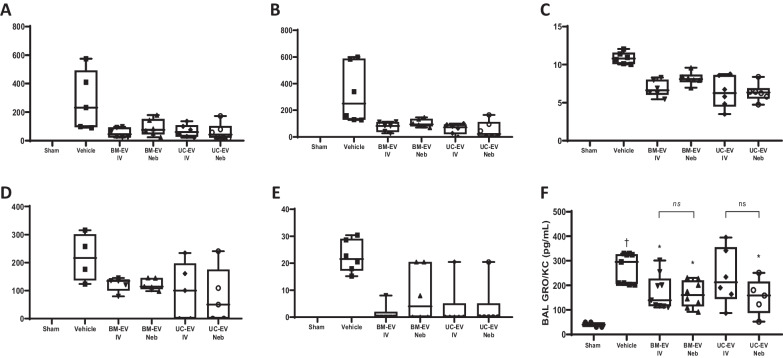
IV and nebulised EV effects on lung inflammatory cytokines in *E. coli* induced pneumonia. Rats received a bolus of *E. coli*, 1 h later were administered BM- or UC-EVs by IV or nebulisation route, and at 48 h cytokines were quantified in BAL by a bead-based multiplex assay. Cytokines assessed included IL-1β **(A)**, IL-6 **(B)**, M-CSF **(C)**, IL-2 **(D)**, IL-5 **(E)** and GRO/KC **(F)**. Sham N = 2–4, Vehicle N = 4–6, BM-EV N = 5–8, Neb BM-EVs N = 5–8, UC-EVs N = 5–8. Neb UC-EVs N = 5–8. † *p* < 0.05 wrt sham. *p < 0.05, **p0.01, ***p < 0.005, wrt pneumonia with vehicle. ns = no significant difference between IV and Neb delivery

Intratracheal installation of *E. coli* increased pro-inflammatory markers such as IL-1β (Fig. 7A), IL-6 (Fig. 7B), macrophage colony-stimulating factor (M-CSF) (Fig. 7C), IL-2 (Fig. 7D), IL-5 (Fig. 7E) and growth-regulated oncogene / keratinocyte chemoattractant (GRO/KC) (Fig. 7F) in BAL compared with the vehicle group. In almost all cases, BM- and UC-EVs significantly reduced cytokine concentrations and this effect was regardless of whether EVs were delivered IV or nebulised directly to the airways. The only outliers observed were IL-2 (Fig. 7D), where BM-EVs significantly reduced the levels regardless the delivery route but only nebulised UC-EVs reached significance on this parameter (Fig. 7F) and UC-EV IV also did not reach significance. Interestingly, nebulised UC-EV still had significant therapeutic efficacy with this parameter (Fig. 7F).

## Discussion

In these studies, we demonstrated that MSC-derived EVs present immunomodulatory and antibacterial capacity, retaining the therapeutic characteristic described for the MSCs cells. We show for first time to our knowledge, the capacity of these EVs to retain comparable therapeutic capacity to non-nebulised EVs after passing through a vibrating mesh nebuliser, opening the possibility of a new delivery route with many advantages over IV delivery. EVs were able to reduce both LPS and *E. coli*-induced lung injury, improving oxygen exchange, reducing physiological and histological damage, meanwhile enhancing bacterial clearance. These effects were observed when EVs were delivered intravenously or nebulised, and findings were largely independent of the EV’s cell source. These findings reinforce the paracrine mechanism of action theory behind MSCs and offers a new therapeutic possibility without the need to use whole cells.

### MSC-EVs retain surface markers and integrity after nebulisation

EV characterisation techniques are still evolving, but their physical parameters and surface markers are the most accepted requirements to determine their cellular origin and purity. We showed that EVs presented the accepted markers CD91, CD61 and CD9 along with the expected MSC specific markers CD146 and CD29 and contained expected anti-inflammatory miRNAs. Nebulisation did not significantly affect the presence of either mIR-146 or miR-181-5p or markers on the membrane surface, suggesting that EVs are either not damaged during transit through the 3-4 µm apertures of the vibrating mesh nebuliser or that they mostly reform after nebulisation. This idea is supported by the change in size observed after nebulisation, probably through EV membranes desegregating on the vibrating mesh and reforming based on their hydrophobic capacity producing small changes on the size. There is also a suggestion of EV aggregation post-nebuliser as some particles size peaks appear to be multiples of single EVs.

### *Nebulised MSC-EVs are immunomodulatory *in vitro

One of the main characteristics required for ARDS therapies is the capacity to mitigate the inflammatory process. In this regard, EVs have been reported to modulate immune cells through miRNA [27], signalling molecules [28] and mitochondrial transfer [5, 11] mechanisms, promoting inflammatory resolution. Nebulised EVs were able to integrate with target lung epithelial cells as well as non-nebulised and were able to mitigate the inflammatory activation caused by LPS in lung cells, restoring cell viability and reducing the production of inflammatory cytokines such as IL-1β and IL-8. Furthermore. Nebulised EVs were able to enhance phagocytosis activity in macrophage-like cells after LPS stimulation, showing that their capacity to interact with immune cells is not diminished by nebulisation, and this might be one of the mechanisms involved in their capacity to reduce pathogen levels. In addition, nebulised EVs showed a capacity to enhance tissue repair.

### Nebulised MSC-EVs have therapeutic effect in pneumonia

Initial IV testing of BM and UC derived EVs demonstrated anti-inflammatory action in an LPS-induced lung injury model. This was evidenced by reduced cytokine and leukocyte concentrations in the lung and gave confidence to progress to a full pneumonia model. Here, rats instilled with *E. coli* presented significant lung injury compared to sham animals, with lower oxygen exchange, increased oedema and inflammatory markers. EVs, whatever the cell source, improved lung function parameters, with no difference on the effect due to delivery route. EVs also attenuated lung oedema, as observed by a reduction in total airspace cell counts and lower fluid levels in the lung overall. This finding could be partially explained by the reduction in the treated animals of neutrophil chemoattractant GRO/KC levels. These results, combined with an increase in the compliance and improvement in lung structure are a robust evaluation of the MSC-EV’s potential in pneumonia therapy. EVs were not only capable of reducing the tissue damage caused by bacterial infection, but also reduced the *E. coli* load in the lungs, and this effect could possibly be explained by the EV’s capacity to enhance phagocytic activity, an effect also retained after nebulisation.

MSCs have been reported to modulate immune cells by a wide variety of contact-dependent and contact-independent mechanisms of action. In our study, we observed that nebulised EVs retain this immunomodulatory capacity. Animals administrated with IV or nebulised EVs from both cell sources had lower levels of pro-inflammatory cytokines such as IL-1β and IL-8, cytokines associated with poor outcome in pneumonia patients, compared to controls. M-CSF is increased in sepsis survivor patients [29] and is associated with monocyte deficiency. EVs were able to reduce M-CSF levels in the lungs compared with vehicle animals, another important mechanistic insight.

## Conclusions

MSCs derived EVs presented anti-inflammatory and pro-healing activity in vitro regardless of the tissue of origin. Nebulisation of these EVs did not affect the described therapeutic capacities. Direct nebulisation of EVs attenuated *E. coli* induced lung damage, reducing bacterial burden and inflammatory markers while improving lung structure and function. These results suggest the possibility of MSCs derived EVs to become a realistic alternative to whole cell therapy, with nebulisation as a new potential delivery route in ARDS patients.

## Supplementary Information

**Additional file 1. Animal procedure schematic.** Animals under anaesthesia received intratracheally an *E.Coli* culture dose establishing the lung injury. One hour later and still under anaesthesia, animals were ventilated using a Flexivent ventilator and EVs or vehicle were delivered using an Aerogen nebuliser connected to the ventilator by a specific module. The system was controlled by a computer allowing to control the delivery internal, and nebulising EVs only in the inspiratory phase to the experimental animals. After this procedure, animals were allowed to recover for the following 48 hours. Finally, animals under anaesthesia were cannulated gaining arterial access and mechanically ventilated to collect different samples such as blood and measure physiological parameters before animals were euthanized.

## Data Availability

All raw data and materials are available on request to the corresponding author.

## Abbreviations

MSC: Mesenchymal stem cell
EV: Extracellular vesicles
*E. coli*: *Escherichia* *coli*
LPS: Lipopolysaccharide
BM: Bone marrow
UC: Umbilical cord
IT: Intratracheally
IV: Intravenously
ARDS: Acute respiratory distress syndrome
ICU: Intensive care unit
CM: Conditioned media
PBS: Phosphate buffered saline
TFF: Tangential flow filtration
NTA: Nanotracking technology analysis
CD: Cluster of differentiation
MTT: (3-(4, 5-Dimethylthiazol-2-yl)-2,5-diphenyltetrazolium bromide
IL: Interleukin
ELISA: Enzyme linked immunosorbant assay
PMA: Phorbol myristate acetate
BAL: Broncheoalveolar lavage
CFU: Colony-forming units
PEEP: Positive end-expiratory pressure
M-CSF: Macrophage colony-stimulating factor
GRO/KC: Growth-regulated oncogene/keratinocyte chemoattractant
