# Supplementary material for: Nebulised mesenchymal stem cell derived extracellular vesicles ameliorate E. coli induced pneumonia in a rodent model
Source: Stem Cell Res Ther. 2023 Jun 6;14:151. doi: 10.1186/s13287-023-03385-6 (PMC10245544; doi:10.1186/s13287-023-03385-6)
Supplement: Supplementary file 1 — Additional file 1. Animal procedure schematic. Animals under anaesthesia received intratracheally an E.Coli culture dose establishing the lung injury. One hour later and still under anaesthesia, animals were ventilated using a Flexivent ventilator and EVs or vehicle were delivered using an Aerogen nebuliser connected to the ventilator by a specific module. The system was controlled by a computer allowing to control the delivery internal, and nebulising EVs only in the inspiratory phase to the experimental animals. After this procedure, animals were allowed to recover for the following 48 hours. Finally, animals under anaesthesia were cannulated gaining arterial access and mechanically ventilated to collect different samples such as blood and measure physiological parameters before animals were euthanized. [file 13287_2023_3385_MOESM1_ESM.docx]

**Supplementary Figure 1**


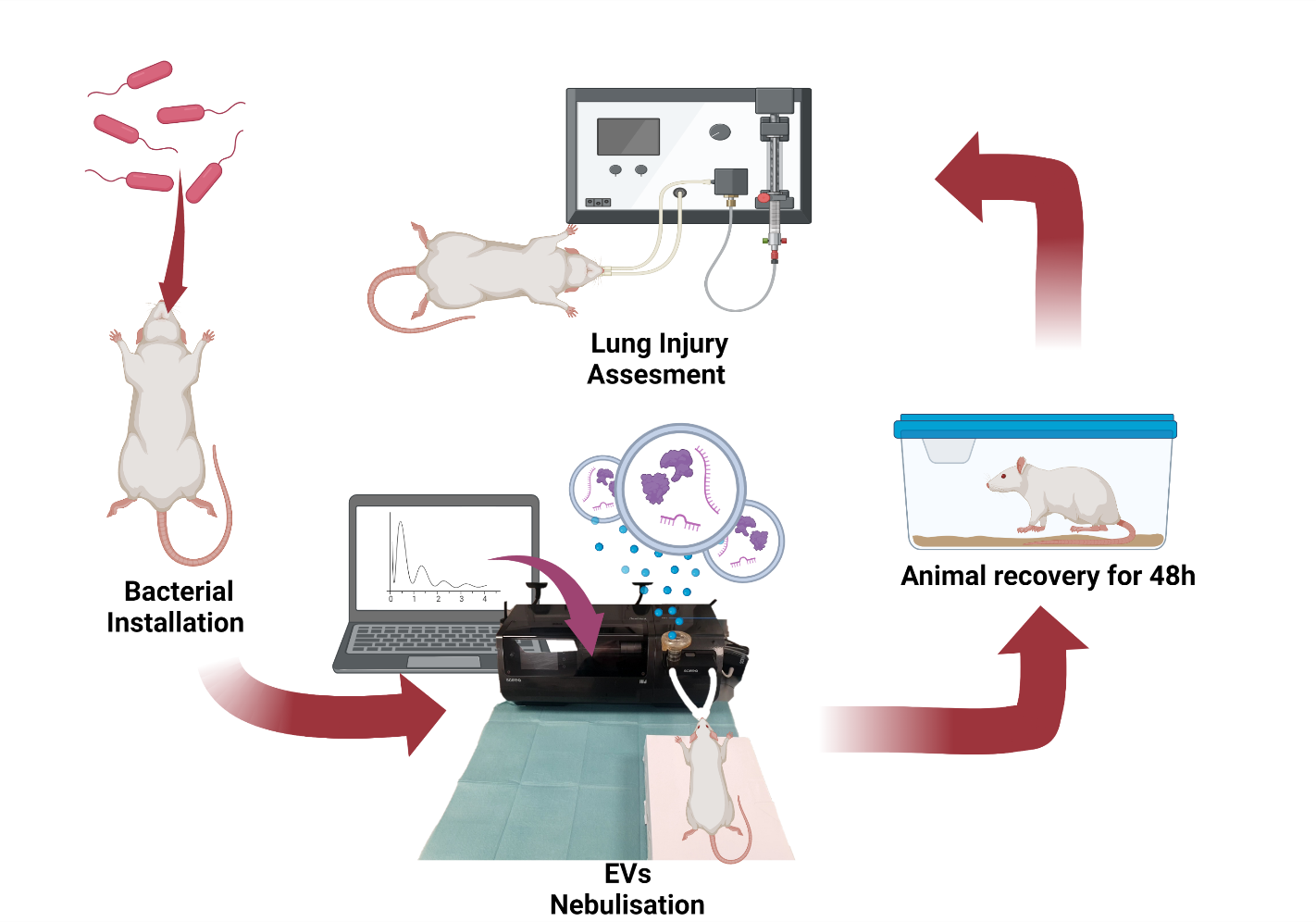


**Animal procedure schematic**

Animals under anaesthesia received intratracheally an *E.Coli* culture dose establishing the lung injury. One hour later and still under anaesthesia, animals were ventilated using a Flexivent ventilator and EVs or vehicle were delivered using an Aerogen nebuliser connected to the ventilator by a specific module. The system was controlled by a computer allowing to control the delivery internal, and nebulising EVs only in the inspiratory phase to the experimental animals. After this procedure, animals were allowed to recover for the following 48 hours. Finally, animals under anaesthesia were cannulated gaining arterial access and mechanically ventilated to collect different samples such as blood and measure physiological parameters before animals were euthanized.
